# Constructing vesicle-based artificial cells with embedded living cells as organelle-like modules

**DOI:** 10.1038/s41598-018-22263-3

**Published:** 2018-03-14

**Authors:** Yuval Elani, Tatiana Trantidou, Douglas Wylie, Linda Dekker, Karen Polizzi, Robert V. Law, Oscar Ces

**Affiliations:** 1 Department of Chemistry, Imperial College London, Exhibition Road, London, SW7 2AZ UK; 2 Department of Life Sciences, Imperial College London, Exhibition Road, London, SW7 2AZ UK; 3 Institute of Chemical Biology, Imperial College London, Exhibition Road, London, SW7 2AZ UK

## Abstract

There is increasing interest in constructing artificial cells by functionalising lipid vesicles with biological and synthetic machinery. Due to their reduced complexity and lack of evolved biochemical pathways, the capabilities of artificial cells are limited in comparison to their biological counterparts. We show that encapsulating living cells in vesicles provides a means for artificial cells to leverage cellular biochemistry, with the encapsulated cells serving organelle-like functions as living modules inside a larger synthetic cell assembly. Using microfluidic technologies to construct such hybrid *cellular bionic* systems, we demonstrate that the vesicle host and the encapsulated cell operate in concert. The external architecture of the vesicle shields the cell from toxic surroundings, while the cell acts as a bioreactor module that processes encapsulated feedstock which is further processed by a synthetic enzymatic metabolism co-encapsulated in the vesicle.

## Introduction

The construction of membrane-encapsulated artificial cells from the bottom up is one of the cornerstone themes in biomimetic biotechnology. One avenue of research centres on functionalising lipid vesicles with biological and synthetic machinery in order to engineer artificial cells that resemble their biological counterparts in form and function^1–6^. Due to their biocompatibility and ability to incorporate biological components to impart function, the potential of vesicle-based artificial cells as soft-matter microdevices is considerable, with applications in directed evolution, protein synthesis, *in vivo* diagnostics, biosensing, drug delivery, and *in situ* drug synthesis^7–15^.

Biological cells, in contrast to their artificial counterparts, have evolved a complex set of biochemical pathways, which makes them capable of dynamic behaviours and of performing an array of tightly regulated functions. They exhibit defined responses to a range of diverse stimuli, and have access to a collection of metabolic pathways. The capabilities of biological cells are thus inherently more advanced than synthetic ones generated from the bottom up.

**Figure 1:**
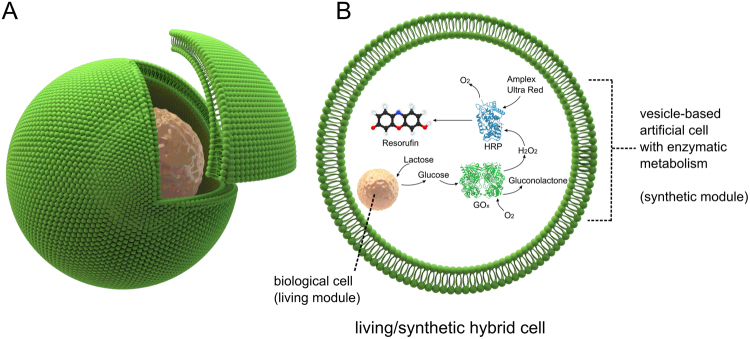
Living/Synthetic hybrid cells. (**A**) Schematic of a biological cell encapsulated inside a vesicle-based artificial cell. (**B**) The encapsulated cell serves an organelle-like function in the vesicle reactor, processing chemical elements which are then further metabolised downstream by a synthetic enzymatic cascade co-encapsulated in the vesicle.

Herein, as a key step to bridge this divide, we present a *cellular bionics* approach where living and non-living components are integrated to yield hybrid systems. We apply this approach to vesicle-based artificial cells: whole biological cells are embedded inside functionalised vesicles for them to perform functions as organelle-like modules. We thus create a new breed of artificial cells that are constructed by fusing cellular and synthetic components in a single self-contained vesicular entity (Fig. 1). Crucially, the encapsulated living cell and the artificial cell host are chemically as well as physically linked together by coupling cellular reactions to enzymatic reactions co-encapsulated inside the vesicle.

Although vesicles have previously been functionalised with biological and synthetic machinery (including membrane channels^15,16^, enzymes^4,17^, DNA origami^18^, quantum dots^19^, and cell-free protein expression systems^20,21^), functionalisation with whole, intact, biological structures (i.e. cells and organelles) has not been achieved. There have been many efforts at encapsulation of cells in droplets^22^, but this is not true of cell-mimetic vesicles. This is an important milestone as vesicles, unlike droplets, have the potential to be used in physiological (aqueous) environments as artificial cells and soft-matter micro-devices with functionalised membranes. The presence of a lipid membrane as an encapsulating shell also paves the way for the incorporation of membrane-embedded machinery (e.g. protein transporters, mechanosensitive channels, photopolymerisable lipids) and for the utilisation of membrane phase behaviour to impart functionality.

Technologies for efficient encapsulation of large, charged chemical species in vesicles have been developed in recent years using the strategy of using water-in-oil droplets as templates around which vesicles are assembled^23–29^. This principle has been extended to encapsulate nano- and micro-sized particles^30,31^, including proteins, beads, and cells, although characterisation of particle encapsulation number and vesicle size distribution was limited. Crucially, these investigations did not involve a demonstration of the use of the encapsulated materials as active functional components in the context of artificial cells.

Others have engineered communication pathways between co-existing populations of biological and artificial cells, an approach which allowed the sensory range of bacteria to be expanded to detect molecules they would otherwise be unable to^32^. A similar effect was achieved by engaging the quorum sensing mechanism of bacteria^33^. However, although these demonstrate the potential of linking artificial cells to biological cells for expanded functionality, there have still not been any demonstrations of living and synthetic cells operating in concert within a single hybrid structure.

In this paper, we develop microfluidic technologies to construct hybrid cells. These are composed of biological cells that serve an organelle-like function, encapsulated in artificial vesicle-based cells. We demonstrate a symbiotic relationship between the vesicle host and encapsulated cell.

We show that the cell is shielded from the external surroundings, and is viable in a solution of Cu^2+^ which would otherwise be toxic. Conversely, we demonstrate that the cell can be used as a bioreactor module to process chemical feedstocks in the vesicle interior. A reaction sequence composed of three cellular and non-cellular steps is employed (Fig. 1), with the cell performing the first step and co-encapsulated enzymes performing the second and third steps. The reaction employed is as follows: (i) hydrolysis of lactose feedstock by the cell to produce galactose, (ii) oxidation of galactose product, yielding H_2_O_2_, (iii) enzymatic oxidation of fluorogenic molecule by H_2_O_2_ for a fluorescent readout. In this way, the encapsulated living cell and the artificial cell host are chemically as well as physically linked together in a cellular bionic system.

## Results and Discussion

### Microfluidic Generation of Hybrids

**Figure 2:**
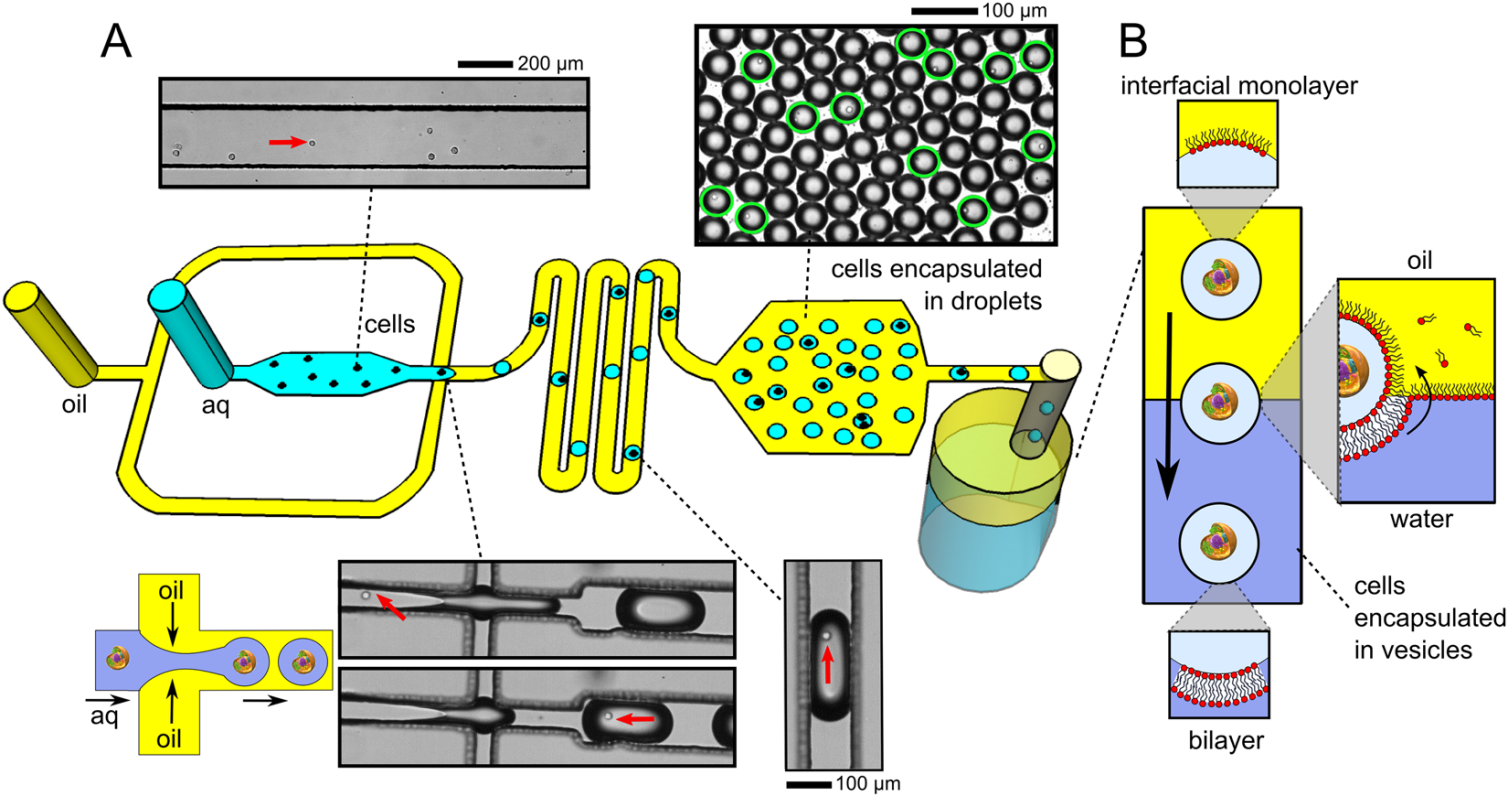
Schematic and microscopy images of the generation of cells-in-vesicles. (**A**) A microfluidic chip was used to encapsulate cells in w/o droplets encased in a lipid monolayer. An aqueous phase containing cells was passed through a flow-focusing junction where it met an oil phase containing lipids, leading to droplet generation. Red arrows show the position of a cell before and after encapsulation. Ratio of cell to droplet size varied from 1:4 to 1:10 depending on the cell type, junction geometry, and flow rates. Droplets were then collected in a chamber where their size and encapsulation number was analysed. A mixture of empty droplets and those containing cells (green circles) were observed. Droplets were then expelled from the device to an emulsion phase transfer column. (**B**) Schematic depicting the transformation of cells-in-droplets to cells-in-vesicles. The droplets descended through the column under gravity. As droplets transferred into the aqueous phase the interfacial monolayer wrapped around them, transforming them into vesicles with cells encapsulated inside.

We construct hybrid cells using droplet microfluidics^34^, enabling the vesicle to have user-defined physical and chemical features and with a defined number of encapsulated cells (see Fig. 2 for schematic and microscopy images; see SI for details). We developed a two-module experimental setup to generate cells-in-droplets which were then converted into vesicles. The first module consisted of a microfluidic chip to generate water-in-oil (w/o) droplets with encapsulated cells. Droplets were generated on-chip in high-throughput (2200 droplets min^−1^) using a hydrodynamic flow focusing geometry, leading to break up of the aqueous phase into discrete w/o droplets. The aqueous channel contained cells in medium (L-15 with 125 mM sucrose), and the oil phase contained dissolved 1-palmitoyl-2-oleoyl-sn-glycero-3-phosphocholine lipids (POPC; 2 mg ml^−1^) in mineral oil, which self-assembled along the water-oil interface, encasing droplets in a monolayer.

Droplets flowed through a serpentine to allow sufficient time for effective droplet stabilisation by a lipid monolayer, otherwise they would merge upon making contact with one another. Droplets then entered an observation chamber for analysis before being expelled from the device, and fed into a second module consisting of an emulsion phase-transfer column, which used the droplets as templates around which lipid vesicles were assembled.

The emulsion phase transfer column consisted of a cylindrical PDMS well on a glass slide with a lower aqueous phase (100 mM glucose in L-15 media) and an upper oil phase (2 mg ml^−1^ POPC in hexadecane). Again, a lipid-monolayer assembled at the interface. Due to the presence of sucrose, droplets generated by the device were denser than the aqueous and water phase of the column which contained glucose. They thus descended through the column under gravity and transferred from the upper oil phase to the lower aqueous phase (Fig. 2B). As they crossed the interface, a second monolayer wrapped around the lipid-encased droplets, resulting in a surrounding bilayer. In this way the w/o droplets were transformed into to w/w vesicles, with the internal contents maintained.

### Cell encapsulation

**Figure 3:**
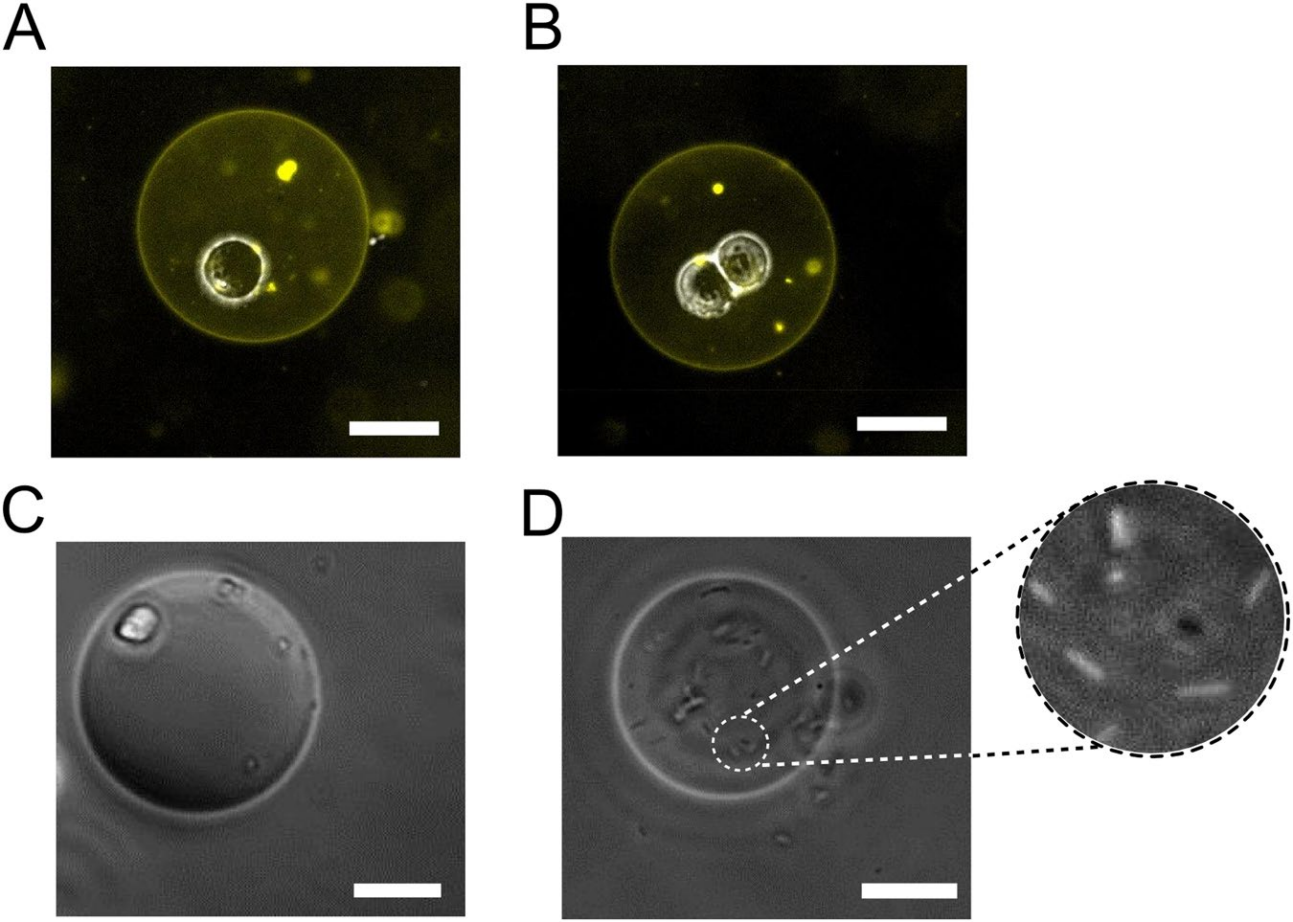
Cells-in-vesicle hybrids. Brightfield/fluorescence composite images of fluorescent POPC vesicles doped with 1 wt.% Rh-PE with (**A**) a single BE cell encapsulated (**B**) two BE cells encapsulated. (**C**) Image of a suspension cell (Toledo B lymphocytes) and (**D**) *E*. *coli* cells encapsulated in a vesicle. Scale bar = 25 µm.

A bacterium and several eukaryotic cell lines were successfully encapsulated in vesicles, with various encapsulation numbers (Fig. 3), including *Escherichia coli* DH5α, BE colon carcinoma cells, HCT colon carcinoma epithelial cells, and suspension Toledo B lymphocyte suspension cells. As they are well characterised, and due to their large size (enabling single-cell encapsulation), BE cells were used for all subsequent investigations unless otherwise specified. To confirm that cells were encapsulated inside vesicles, we added a 1 wt.% fluorescent lipid, leading to a clear fluorescent boundary at the vesicle edges. For controlled encapsulation of the eukaryotic cells, strategies to prevent cell aggregation prior to droplet generation had to be implemented, including adding EDTA (3 mM) to the buffer to prevent cation-dependent cell-cell adhesion, and a mesh to filter out aggregated cells before insertion in the microfluidic chip.

Despite the mechanical shear forces associated with droplet generation, cell flow, and ejection from a microfluidic device, the encapsulated cells were found to be viable (97%; n = 53) and metabolically active (82%). This was determined by a DNA binding assay to detect membrane integrity (NucGreen) and a mitochondria and cytoplasm redox potential assay^35,36^ (alamarBlue), respectively.

**Figure 4:**
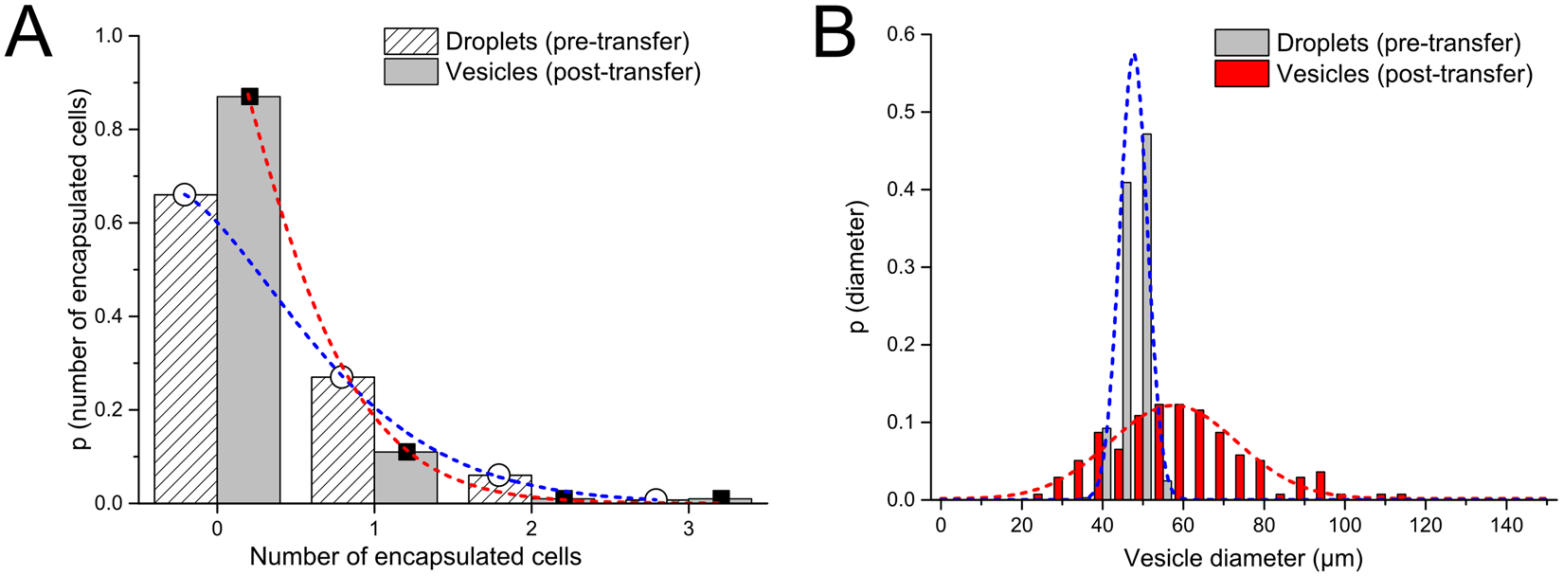
Cell encapsulation and vesicle size distribution (**A**) Graph of number of cells encapsulated in the droplet precursors and in the vesicles. These followed a Poisson distribution (dotted lines) where λ = 0.41, 0.13 respectively. (**B**) Size distribution of the droplet precursors and vesicles. These followed Gaussian distributions (dotted lines).

The use of droplet microfluidics enables droplets of defined volumes to be generated, determined by the device geometry and flow rates. Using our design droplets with an average radius of 22 μm (45 pL) were generated, with a narrow size distribution of 7% c.v (Fig. 4). The processes of transforming droplets to vesicles led to an increased mean radius of 29 μm (102 pL) and a wider size distribution of 30% c.v. Due to the stochastic distribution of cells in the aqueous channel before they enter the droplet generation region, the number of cells encapsulated in droplets follows a Poisson distribution with λ = 0.41 (n = 461), which changes to λ = 0.13 (n = 193) as droplets are transformed into vesicles (see SI S8 for further discussion).

### Encapsulated cell as an organelle-like reactor

**Figure 5:**
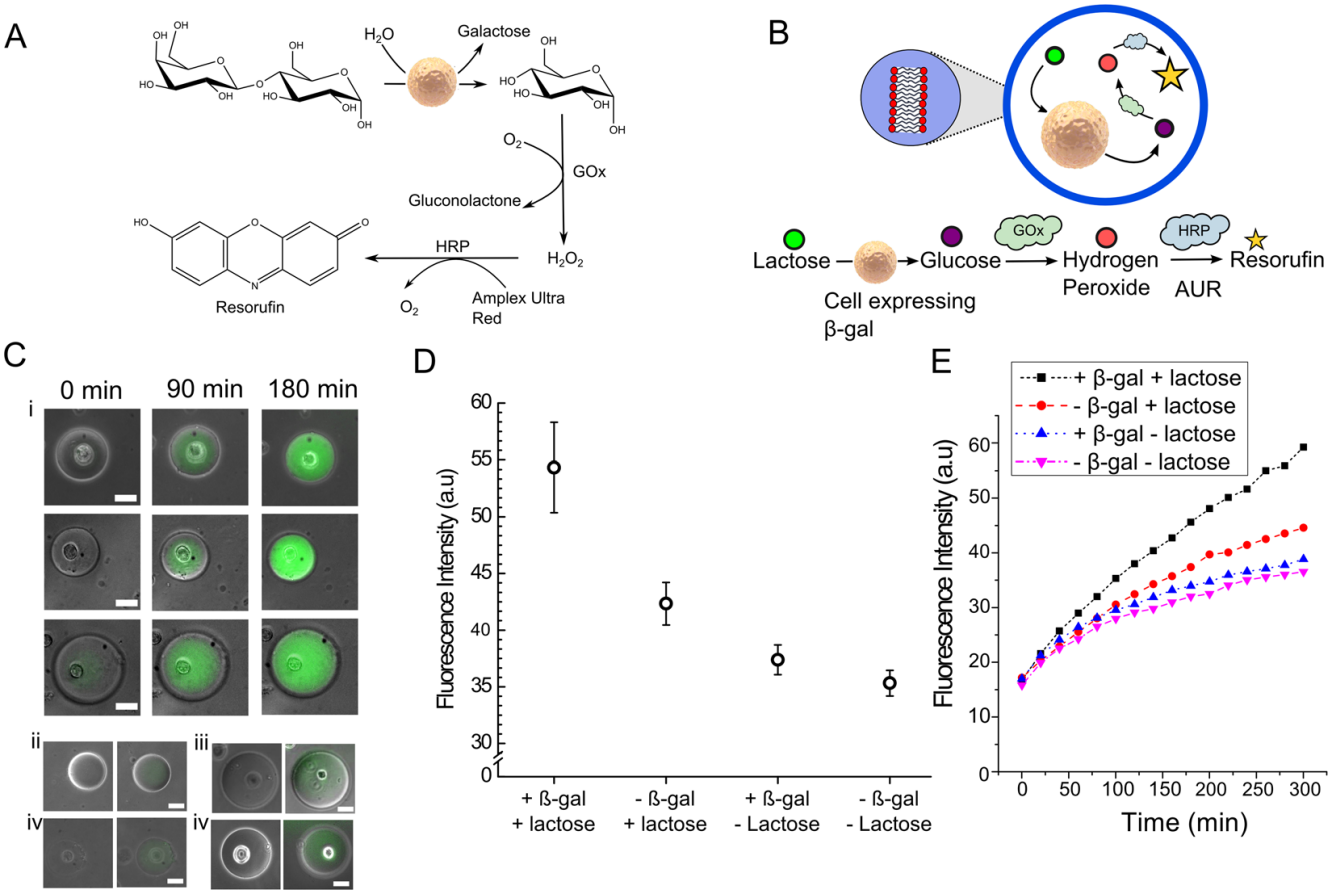
Encapsulated cell as an organelle-like bioreactor. (**A**) Reaction scheme (**B**) Vesicle/cell hybrids were engineered where encapsulated cells performed one step of a multi-step enzymatic pathway (hydrolysis of lactose to glucose). Glucose was then further metabolised in the vesicle interior by an artificial enzymatic cascade, resulting in a fluorescent product (resorufin). (**C**) (i) Representative brightfield/fluorescence composite images of vesicles/cell hybrids at different time points, showing successful synthesis of reaction products over time compared to control experiments (ii) with no encapsulated cell, which exhibited minimal fluorescence after 180 minutes. Scale bar = 25 µm. (**D**) Graph demonstrating mean fluorescence of vesicle/cell hybrids with a transfected cell and encapsulated feedstocks 180 minutes after generation, as well as three control scenarios. Error bars represent standard deviations of mean fluorescence after 180 minutes from three independent preparation runs (**E**) Kinetics trace of bulk fluorescent levels after three hours of the cascade reaction. The presence of transfected cells significantly increased reaction yield, confirming the successful coupling of the cellular pathway to the artificially added elements in the cascade.

Constructing vesicle/cell hybrids raises the possibility of using the encapsulated cells as a bioreactor modules within vesicle-based artificial cells. This would allow the coupling of cellular to non-cellular pathways co-encapsulated in the vesicle. To demonstrate this, we devised a three-step biochemical pathway, with the first step being performed by the encapsulated cell, and the final two steps performed externally from the cell by enzymes present in the vesicle interior (Fig. 5). The first step was the hydrolysis of lactose to glucose and galactose by β-Galactosidase (β-gal) expressed by an engineered BE cell designed to express β-gal. This was followed by translocation of glucose out of the cell, and its subsequent oxidation by Glucose Oxidase (GOx) in the vesicle interior, producing H_2_O_2_. The final step was oxidation of Amplex Ultra Red (AUR) by H_2_O_2_ in the presence of horseradish peroxidase (HRP) to yield the fluorescent resorufin end product. The use of AUR as opposed to its analogue, Amplex Red, was due to its membrane impermeability^37,38^, allowing its accumulation^39^ to be monitored in the vesicle.

Vesicle/cell hybrids were generated as before, but with transfected cells, GOx, HRP and AUR encapsulated in the vesicles, and fluorescence monitored via fluorescence microscopy (Fig. 5C). Over a period of 180 minutes fluorescence significantly increased over time compared to the control scenario where vesicles did not contain any cells. Bulk measurements, conducted using a fluorimeter on a system containing both cellular and synthetic components, revealed a 3.5 fold increase in fluorescence over 300 minutes, further confirming the coupled reaction cascade was operational (Fig. 5D,E). This is a significant increase compared to the control scenarios where vesicles contained cells not expressing β-Gal or/and no lactose feedstock (unpaired t-test p-values of 0.010, 0.002, 0.001 respectively; significance threshold was set at 0.05). The control experiments also revealed an increase over time (albeit a substantially reduced, ~2 fold increase). This was attributed to trace amounts of lactose and galactose remaining in the medium, photo-activated oxidation of AUR and non- β-gal mediated breakdown of lactose by the cell.

### Cell shielding by vesicle host

**Figure 6:**
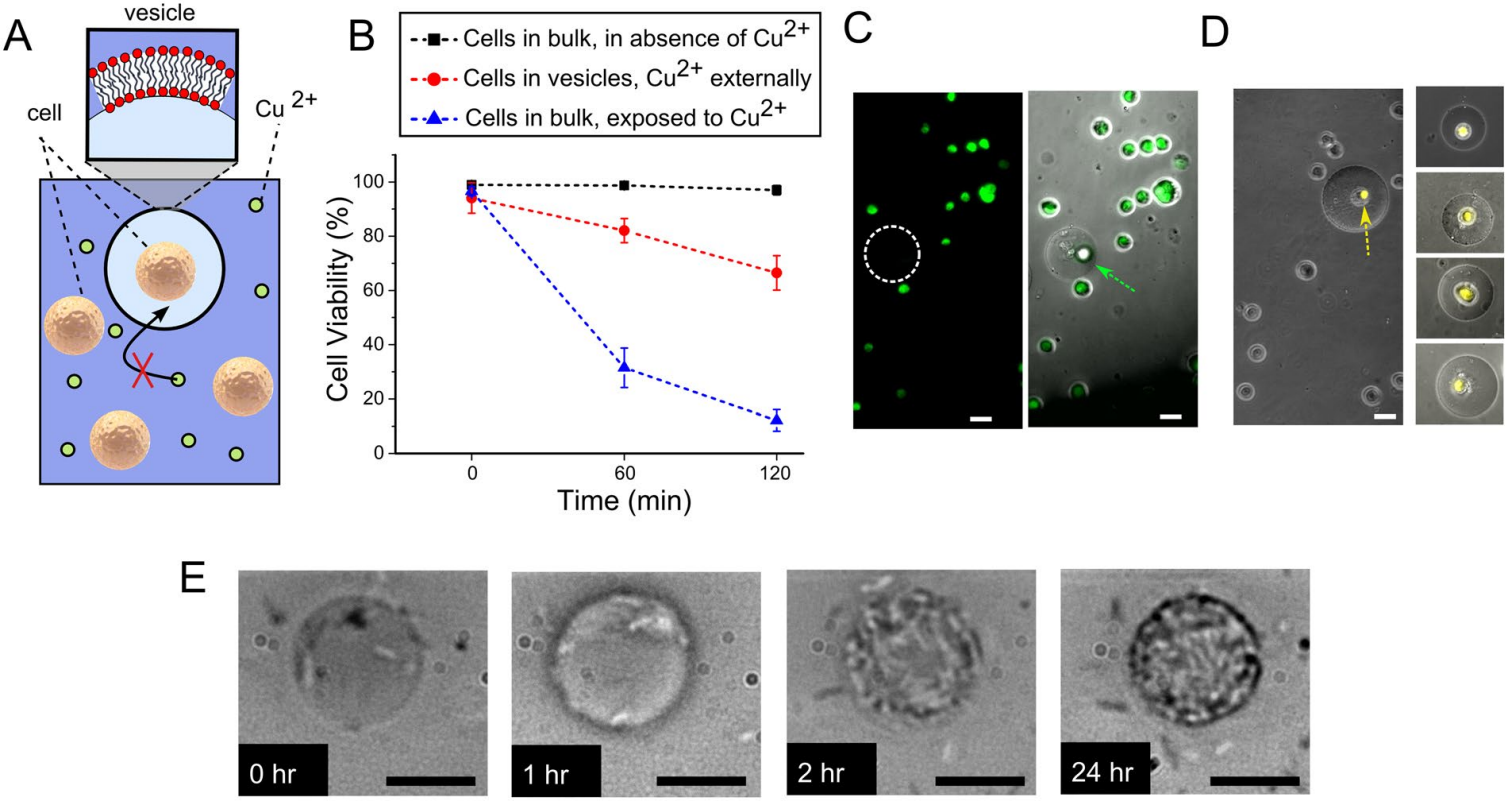
Vesicle shielding of cells from toxic surroundings and cellular replication in vesicles. (**A**) Schematic of assay; Cu^2+^ is permeable to vesicles and is unable to penetrate the membrane and cause cell death. (**B**) Graph showing the viability of cells over time in a variety of conditions. Error bars represent standard deviations from three independent trials. (**C**) Fluorescence and brightfield/composite image from a viability assay of cells-in-vesicles with Cu^2+^ in the external solution. Dotted circle represents the location of GUV in the fluorescent channel. Encapsulated cells were viable (arrow) while unencapsulated cells were non-viable (green channel). (**D**) Composite fluorescence/brightfield image from a metabolic activity assay with Cu^2+^ in the external environment. Cells encapsulated in vesicles remained metabolically active (yellow channel and arrow) despite the presence of the toxic species. (**E**) *E*. *coli* cells encapsulated in POPC vesicles were seen to replicate and increase in number over a period of 24 hours, demonstrated cellular viability over time in the confined environment. Scale bar = 25 µm for all images.

One prospective application of hybrid artificial cells relates to the vesicles capacity to shield its interior including the real cell from the external environment, which may be toxic. We explored this using a fluorescence assay to monitor the viability of cell-in-vesicles with Cu^2+^ present in the external solution (Fig. 6). Cu^2+^ was present at levels known to lead to cell death due to initiation of oxidative damage and interference with important cellular events^40^.

Cells were generated as before, but with a passivating layer of BSA on the glass substrate to prevent ion-mediated vesicle rupture on the surface. A viability reagent (NucGreen) was present in both the internal and external vesicle solutions, and CuCl_2_ was added to the external solution after generation to a final concentration of 5 mM. Fluorescence was monitored over a period of 120 minutes to determine levels of cell death (n > 120). Over 66% of cells-in-vesicles were viable, compared to 6% in the control scenario of unencapsulated cells, a statistically significant difference (unpaired t-test p-value = 0.0002), indicating that the vesicle effectively shielded the cell from membrane impermeable species^41,42^. Cells-in-vesicles had lower viability than those present in bulk solution without the presence of copper in identical conditions (98% viability), presumably due to the effects of confinement, including the build-up of waste products in the vesicle interior^43^. This limitation can be addressed in future through the addition of selective protein pores that allow the expulsion of waste products, but prevent the inflow of harmful materials. The lack of influx of Cu^2+^ to the POPC GUV interior was confirmed through a permeation assay involving the Cu^2+^ sensitive dye calcein (see SI 9).

We note that the ability of vesicles to shield cells is highly dependent on the physicochemical properties of the molecule in question (e.g. the LogP), and of the permeability the vesicle membrane itself^44,45^. Large and/or charged biomolecules (e.g. enzymes, nucleic acids, DNA)^46^ are largely impermeable to phospholipids which makes this system particularly useful in physiological settings.

### Longevity of hybrid cells

A key indicator of cellular activity is their ability to replicate, a metric which was used to assess the potentially deleterious effects of confinement. Cells-in-vesicles were monitored over a period of 24 hours. Encapsulated *E*. *coli* were seen to increase in number over this time (Fig. 6E). This ability to replicate shows that the effects of confinement, potential nutrient deprivation, and stress resulting from microfluidic generation do not significantly interfere with cell biology in this case. However, replication was not observed with the eukaryotic cell-lines despite them being metabolically active. This could be due to the higher sensitivity and reduced robustness of these cells compared to *E*. *coli*, which could lead, for example, to an arrest in the cell cycle and potential confinement-induced genetic re-programming via cellular self-signalling, as have been observed in other systems^47^. Their effective concentration could also mean that they are also close to the cell growth curve plateau^43^. The presence of cells inside the vesicles did not affect the stability or integrity of the membrane. The vesicle remained intact a week after generation, and still successfully carried encapsulated cargo over this time (SI S8).

### Summary and Conclusion

We have developed and characterised microfluidic technologies to encapsulate biological cells in vesicle-based artificial cells, thus creating hybrid structures composed of synthetic and cellular modules. The use of microfluidics allows the design of hybrids with user engineered features. Tuning droplet features using microfluidics is well-established by altering parameters such as device geometries and flow rates^34^, and as droplets act as templates around which vesicles are assembled, these features are transferred to the vesicle in turn. Hybrid cells were produced at high throughput and with control over vesicle size, biomolecular content, and cell encapsulation number. The experimental setup also exposes the technique to varied advantages of microfluidic technologies, including low sample size, increased efficiency, and amenability to multiplexing, automation, and translation into portable lab-on-chip devices^29,48^.

A conceptual parallel can be drawn between hybrid cells constructed here and eukaryotic cells, which originated through an evolutionary process of endosymbiosis where formerly free-living organisms were subsumed into their host. Likewise, the encapsulated cell and vesicle-based artificial cell host exhibit a mutually beneficial relationship: the vesicle shields the cell from toxic surrounding, and the cell provides the vesicles access to cellular biochemistry in a distinct organelle-like sub-compartment.

Viewed in the context of vesicle-based artificial cells, the encapsulation of biological cells allows them to serve modular functions. In the experiments above, the cell performed one step of a reaction sequence, with subsequent steps carried out by non-cellular components, thus combining cellular biochemistry with artificially devised chemistry in a compartmentalised system. The fact that vesicles remained intact days after they were generated, and cells were viable and able to reproduce inside the confined environment, further cements the potential of such hybrid systems.

This approach paves the way for the richness and versatility of cell biology to be interfaced with artificial cells generated from the bottom up, which could in turn lead a new generation of functional devices and materials. Such systems could find applications in cell therapy^49^, in chemo-enzymatic hybrid cascades^50^, in the study of confinement on biological systems, and in cell-based sensors.

## Materials and Methods

Full experimental details are provided in the Supporting Information. Briefly, cells-in-vesicles were prepared using a PDMS-based microfluidic device that was fabricated using standard soft-lithography techniques. Water-in-oil droplets were formed where the oil phase consisted of 2 mg ml^−1^ POPC in mineral oil and the water droplets contained BE cells (ca. 4 × 10^7^ cells ml^−1^; L-15 media; 3 mM EDTA). Droplets were then converted into vesicles using the emulsion phase transfer method. This method relies on a density difference between the vesicle interior and exterior in order for vesicles to descend through the column under gravity. As such, vesicle interior contained sucrose (125 mM) and vesicle exterior contained glucose (100 mM), with the vesicles withstanding the resulting slight osmotic imbalance.

Unless otherwise noted, BE human colon carcinoma cells were used and cultured using Dulbecco’s Modified Eagles Medium (DMEM, no glucose) and 10% (v/v) foetal bovine serum (FBS) in a cell incubator. Other cells successfully encapsulated were HCT colorectal carcinoma cells, suspension Toledo B lymphocyte suspension cells, and *E. coli.* When conducting experiments cells were harvested up to four days following plating, and kept in L-15 media. In vesicle bioreactor experiments cells were engineered to express β-gal by transfection with Lipofectamine 3000, pSV-β-Galactosidase transfection vector, and Opti-MEM media, according to the supplier’s protocol.

For the organelle bioreactor experiments, BE cells expressing β-gal were mixed 1:1 with a reaction mix containing 125 mM sucrose, 100 µM lactose, 100 µM AUR, 2 U ml^−1^ GOx, and 0.2 U ml^−1^ HRP. Fluorescence levels were monitored over time on a fluorimeter (Cary Eclipse; λex = 572 nm, λem = 583 nm). For fluorescence microscopy experiments, cells-in-vesicles were generated with the above reagent mix encapsulated in the vesicle interior, and monitored with 100 ms exposure. In the control experiments, lactose was absent from the reagent mix, and non-transfected cells were used.

## Electronic supplementary material

Supplementary Information
