# Supplementary material for: Constructing vesicle-based artificial cells with embedded living cells as organelle-like modules
Source: Sci Rep. 2018 Mar 14;8:4564. doi: 10.1038/s41598-018-22263-3 (PMC5852042; doi:10.1038/s41598-018-22263-3)
Supplement: Supplementary file 1 — Supplementary Information [file 41598_2018_22263_MOESM1_ESM.pdf]

Supplementary Information

Constructing vesicle-based artificial cells with embedded living cells as organelle-like modules

Yuval Elani*, Tatiana Trantidou, Douglas Wylie, Linda Dekker, Karen Polizzi, Robert V. Law and Oscar Ces*

Contact: yuval.elani10@imperial.ac.uk ; o.ces@imperial.ac.uk

S1. Lipid-in-oil preparation

Lipids were purchased from Avanti Polar Lipids, USA. All vesicles were composed of POPC, apart from fluorescent vesicles which contained 1 wt. % 1,2-dioleoyl-sn-glycero-3-phosphoethanolamine-N-(lissamine rhodamine B sulfonyl) (ammonium salt) (Rh-PE). Lipid-in-oil was prepared by dissolving 6 mg of lipid in 200 µl of chloroform, removing chloroform under a nitrogen stream to create a lipid film, and leaving in lyophiliser for over two hours to remove excess solvent. Mineral oil or hexadecane (Sigma Aldrich, USA) was then added to make it up to 2 mg ml^{-1}, and the solution was sonicated for 60 minutes at 50° C to dissolve the lipid.

S2: Microfluidic device design, fabrication and operation

The PDMS-based device was constructed using standard soft lithography techniques, using a silicon wafer and Su-8 3050 (Microchem, USA) as a photoresist to create a master. PDMS (Sylguard 184, Dow Corning, USA) was mixed at a 9:1 ratio of curing agent to elastomer, mixed thoroughly, poured over the master, degassed and cured at 60°C overnight. Cured PDMS was then peeled off revealing device features. 0.5 mm radius access holes were drilled in the inlets and outlets, and the device sealed by bonding to a second 1 mm thick PDMS sheet by exposing both to plasma for 30 s and firmly pressing them together. Tubing (Harvard Apparatus, USA; polyethylene, 1 mm OD) were inserted into inlets and outlets at one end, and to 1 ml syringes on the other. The syringes were connected to precision syringe pumps (Fusion 100, Chemyx, USA) to drive flow. Immediately before operation of the device, the cell solution was well agitated and filtered through a nylon mesh (40 µm pores; BD Falcon, UK) to remove any cell aggregates.

The device was designed so that cells entered the device through a wide (200 µm) inlet channel, to prevent cells clumping and clogging the channel. Cells then entered a narrow cross-shaped flow-focusing junction (45 µm x 45 µm), where droplets with these characteristic dimensions were generated. Droplets then flowed through a meander (c. 90 µm width; 50 mm total length) to give sufficient time for effective stabilisation by a lipid monolayer, else they would merge when droplets made contact with one another. The inlet channel was wider than downstream channels (200 µm vs 90 µm) to prevent cell accumulation and blockages before droplet generation. Droplets finally entered a wide observation chamber for analysis (c. 2.5mm x 2.5 mm) and were then expelled from the device.

Pump flow rate were set at 8 µl min^{-1} for the lipid-in-oil phase (hexadecane) and 1 µl min^{-1} for the aqueous phase containing BE cells (ca. 4×10^7 cells ml^{-1} ; L-15 media). 3 mM EDTA was also present to prevent cation-dependent cell-cell adhesion. Once droplets were generated, they were expelled from the chip into the phase transfer column via 50 mm of tubing, where they descended under gravity and transformed into vesicles.

S3. Column preparation and emulsion phase transfer

To generate vesicles from droplet precursors a water/oil column for vesicle generation was prepared in a cylindrical PDMS well (10 mm diameter, 10 mm depth) with a glass slide as a substrate. First, to prevent unwanted vesicle/glass interactions, BSA (1 wt % in DI water, 100 µl; Sigma-Aldrich, USA) was deposited on the glass surface, the well placed in a 50 °C oven overnight for water to evaporate, and excess BSA removed with DI water to leave behind a passivating monolayer. The lower column aqueous phase (100 mM glucose in L-15 media, 200 µl) was added followed by upper lipid-in-oil phase (mineral oil, 200 µl). The column was left to stabilise for at least one hour allowing a well-packed interfacial lipid monolayer to assemble at the interface, after which time droplets were added. This was done by placing the outflow tubing directly into the top portion of the oil phase to prevent potential disruption of the column water/oil interface; the tubing was held in place using a clamp and a hoffman clip. The outflow tubing was only inserted into the column after flow rates stabilised and monodisperse droplets were being generated on the chip. It was left in place for ca. 5 minutes before being removed, with approximately 45 µl of emulsion solution being expelled during this time.

In the microscopy experiments, the column was imaged directly imaged after vesicles sedimented on the substrate (ca. 5-10 minutes after on-chip co-encapsulation). In all other experiments, vesicles were collected by first removing the oil phase with a syringe (carefully ensuring no oil film/droplets remained), and then removing the aqueous phase with a pipette. By comparing the number of encapsulated cells to the number of cells-in-vesicles, the efficiency of the phase transfer process was estimated to be below 10 %, although we note that this can be improved upon by using fully integrated microfluidic devices in future.

S4. Imaging and vesicle characterisation

Microscopy images were obtained with an inverted microscope (Olympus IX81), a mercury arc lamp for fluorescence (X-Cite 120, EXFO photonic solutions), and CCD camera for detection (Retiga EXi, QImaging). A TRITC filter used to image resorufin, Rh-PE, and alamarBlue and FITC filter for NucGreen. To quantify encapsulation number and size droplets and vesicles, image analysis software and thresholding functions were used (ImageJ, NIH). To assess vesicle viability and metabolic activity, NucGreen and AlamarBlue stains were used (Thermo Fisher; 10 % v/v with cell-suspension media) and fluorescence levels measured (500 ms exposure) and compared against a known population of living and dead cells as controls for the threshold. In the copper toxicity experiments, copper was present in the bulk external solution as 5 mM $CuCl_2$ (Sigma-Aldrich, UK) and cells-in-vesicles were generated as before, but with an absence of EDTA . NucGreen reagent was also present in the internal vesicle volume and external aqueous solutions (10 % v/v).

S5. Cell culture procedures

Unless otherwise noted, BE human colon carcinoma cells were used and cultures using Dulbecco's Modified Eagles Medium (DMEM, no glucose; Thermo Fisher) and 10 % (v/v) foetal bovine serum (FBS; Sigma-Aldrich) in a cell incubator. Cells were split 1:4 approximately every 4 days using Accutase (Sigma-Aldrich) to detach cells. Other cells successfully encapsulated were HCT colorectal carcinoma cells and suspension Toledo B lymphocyte suspension cells. When conducting experiments cells were harvested up to four days following plating, and kept in L-15 media. As FBS contains some degree of sugars inside (e.g. lactose and galactose), as much of this as possible was removed to limit the effect of false positives in the cell/enzymatic cascade experiments. Residual FBS and DMEM media were removed by gentle centrifugation at 400 g for 2 minutes at and re-suspension in L-15 media, a process that was repeated twice.

In vesicle bioreactor experiments cells were engineered to express β-gal by transfection with Lipofectamine 3000 (Invitrogen), pSV-β-Galactosidase transfection vector (Promega), and Opti-MEM media (Thermo Fisher), according to the supplier's protocol.

Successful transfection was confirmed using an ImaGene Green Assay (Thermo Fisher Scientific). This assay uses the lipophilic probe C_{12}FDG, which is based on the molecule fluorescein di-β-D-galactopyranoside. This molecule gets hydrolysed by β-galactosidase to yield a fluorescent product which is retained in the cells as it contains a 12-carbon lipophilic moiety. The probe was introduced into the cells 24 hours after transfection. To do this, we removed the medium from confluent cells, covered with 33 μM of the probe solution in medium, and incubated for 60 minutes. Cells were then detached from the surface using Accutase, pelleted as before, re-suspended in fresh media, and observed under the microscope (FITC filter, 500 ms exposure). As can be seen in Figure SI 1 below, fluorescence was observed in transfected cells (transfection efficiencies > 90%, n = 189). With non-transfected cells, used as a control, false positives were observed at a rate of < 5% (n= 223).

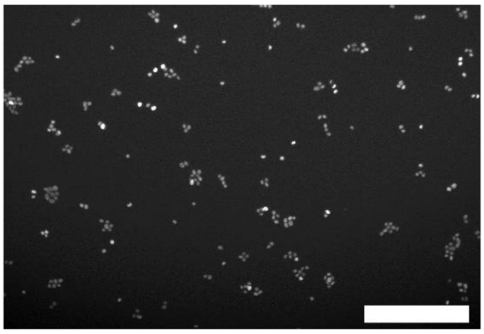

Figure SI 1. Fluorescence microscopy images of transfected cells incubated in fluorogenic C_{12}FDG. A > 90% transfection efficiency was recorded. Scale bar = 200 μm.

S6. Bacterial growth conditions

A single colony of *E. coli* were used to inoculate 2 mL of LB medium and grown for approximately 6 h during the day at 37 °C with shaking at 250 rpm. The starter culture was diluted 1:100 into 5 mL of M9 minimal medium (1x M9 salts, 2 mM $MgSO_4$, 0.1 mM $CaCl_2$, 0.34 g/L thiamine hydrochloride, 1 g/L NH_4Cl, and 0.4% glycerol) and grown overnight at 37 °C with shaking. The next morning, 1 mL of the culture was centrifuged for 3 minutes at 10, 000 g, the supernatant discarded, and the bacterial pellet resuspended in 1 mL of culture.

S7 Organelle bioreactor assay

Hybrid cells were constructed which contained in their interior: BE cells expressing B-gal; GOx; HRP; AUR. The vesicle membrane was composed of POPC. L-15 buffer was present both internally and externally to the vesicle. To enable phase transfer generation of vesicles, sucrose (125 mM) was present in the vesicle interior, and glucose (100 mM) was present in the exterior solution. For the cascade performed in bulk, 100 μl of transfected cells (ca. 4×10^7 cells ml^{-1}, L-15 media) were added to 100 μL of reaction mix (125 mM sucrose, 100 μM lactose, 100 μM AUR, 2 U ml^{-1} GOx, and 0.2 U ml^{-1} HRP in PBS; Thermo Fisher). Fluorescence levels were monitored over time on a Cary Eclipse fluorimeter, λex = 572 nm, λem = 583 nm, ex/em slit = 5nm, PMT = 500 V, averaging time = 200 ms. 100 μL of sample was added to a 96 well-plate and recordings were taken every 20 minutes, with data acquisition beginning precisely 10 minutes after sample preparation (defined as t = 0). Three replicates were run for each condition, where each replicate represented an individual hybrid cascade preparation run. The background fluorescence was taken to be the value of an identical mixture in the absence of cells at any given time point. This value was subtracted from the signal, with no further data processing or

normalisation. For fluorescence microscopy experiments, cells-in-vesicles were generated using the chip, with the reagent mix encapsulated in the vesicle interior, and monitored with 100 ms exposure. Device flow rates were kept constant throughout (9 µl min^{-1} in total), and the device was left producing droplets for 5 minutes before being stopped to ensure the number of vesicles generated per experimental run remained constant.

S8: Characterisation of cells-in-vesicle hybrids

Due the stochastic distribution of cells in the aqueous channel before they enter the droplet generation region, the number of cells encapsulated in droplets follows a Poisson distribution, λ= 0.41, n= 461, where 66% of droplets were empty, 27% had a single encapsulated cell and 7% had two or more encapsulated cells. Previous studies have shown that a Poisson distribution is expected.[1,2]
The process of droplet transformation into vesicles altered this distribution (λ= 0.13, n= 193). Figure SI 2A shows a typical microscopy image that was recorded post vesicle generation, showing that the majority of vesicles were empty. Figure SI 2B shows several vesicles with single encapsulated cells, obtained from different frames of the same sample.

The shift in distribution during vesicle generation has been observed by others, and may be due to an interplay between a number of factors. First, not all droplet survive the phase transfer process – some simply merge with the surrounding aqueous phase before transforming into vesicles. The efficiency of this process is known to be a size-dependant [3-5], with the probabilities of larger droplets successfully transferring being greater than smaller droplets. It is also likely that cells have a destabilising effect on the interface, leading to lower transfer efficiencies at higher encapsulation numbers. A second factor is that before transforming into vesicles some droplets merge together to form larger droplets, while others split up. These processes are likely to occur at the chip-to-outlet interface, where droplet accumulate, and high shear forces are exerted. Finally, it is possible that droplet shrinking via dissolution into the oil was occurring prior to vesicle generation.[6,7]

Although the vesicle size distribution was broader than the starting droplet distribution, it is still narrower than with alternative generation strategies.[8] The ratio between the droplet precursors and vesicle dimensions is likely to converge with the development fully integrated devices[9-11] employing either passive methods[12,13] or active cell sorting techniques.[14]

We also observed that vesicles remained intact with the encapsulated material more than a week after they were first formed. This can be seen by microscopy images of cells-in-vesicles that were stored at room temperature and imaged seven days after formation (Figure SI 2C). As expected however, the morphology of the cells indicate that they were no longer viable after this time. Statistical analysis of this sample revealed that after seven days the proportion of vesicles containing cells was similar to those imaged immediately after generation (11 % and 12 % respectively, n > 150), which further indicates that the presence of cells did not lead to destabilisation or rupture of the vesicles.

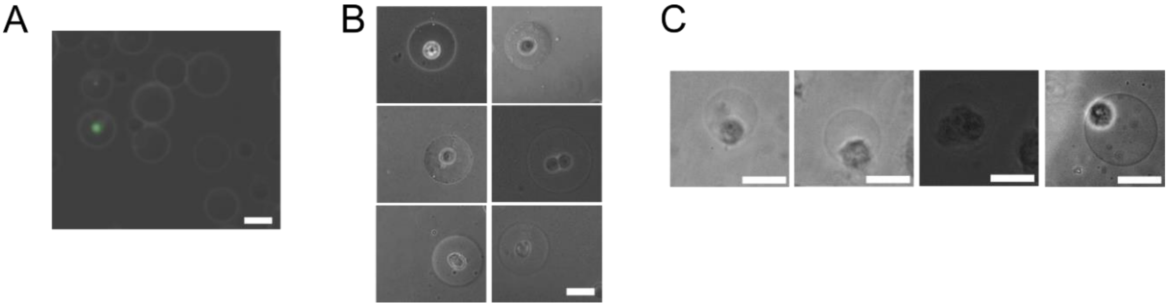

Figure SI 2. (A) Microscopy image of a typical frame after phase transfer, with the encapsulated cell stained with an alamarBlue viability reagent for clarity, demonstrating that the majority of vesicles are empty. **(B)** Images of several vesicles with encapsulated cells, obtained from different frames. **(C)** Images of cells-in-vesicles seven days after generation. Scale bars = 25 µm.

S9: Permeability of POPC GUVs to $CuCl_2$

Shielding of cells within POPC GUVs from Cu^{2+} in the vesicle exterior relied on the lack of permeation of Cu^{2+} through the vesicle membrane. To demonstrate that this we performed a leakage assay involving a Cu^{2+} sensitive dye (calcein).[15] First, we performed a calibration curve to show that 1 mM calcein in quenched at 5 µM Cu^{2+} and above (Figure SI 3A). Fluorescence (ex = 495 nm, em = 515 nm) of calcein at different concentration of $CuCl_2$ in buffer (HEPES, 125 mM sucrose) was recorded on a fluorimeter.

Next, we performed a Cu^{2+} membrane permeation assay where 1 mM calcein was encapsulated in POPC GUVs in HEPES buffered saline (see Figure SI 3B for assay schematic). Vesicles were formed emulsion phase transfer using a procedure described elsewhere.[16,17] Unencapsulated calcein was removed by centrifuging the vesicles to form a pellet (6000 g, 10 minutes), removing the supernatant, and resuspending in fresh buffer; this was repeated three times. $CuCl_2$ was added to the GUV exterior to yield a final concentration of 5 mM, and the fluorescence of 100 µl of sample monitored over time on a fluorimeter. Over 200 min, no significant change in fluorescence was observed, indicating minimal influx of Cu^{2+} ions (Figure SI 3C, D). When vesicles were ruptured through the addition of 1 µl of 8 wt.% Triton X-100 in DI water, a c. 100 fold drop in fluorescence was observed as Cu^{2+} was able to access the dye. This shows that the system was operational. As a control, when 5 mM Cu^{2+} was present both inside and outside GUVs, minimal fluorescence was seen, demonstrating successful quenching at the theoretical endpoint, if Cu^{2+} were to fully permeate to the GUV interior; this signal did not change when vesicles were ruptured. As a second control, an experiment was run with no Cu^{2+} either inside or outside the GUVs. In this scenario, no quenching was seen either before or after rupture.

These experiments demonstrated at the timescale in which our cells-in-GUV assays were run (120 min) negligible (< 0.1 %) Cu^{2+} permeation into the GUV membrane occurred. This is confirmed by results of the cell assay, which showed that cells-in-GUVs were viable in a 5 mM Cu^{2+} solution.

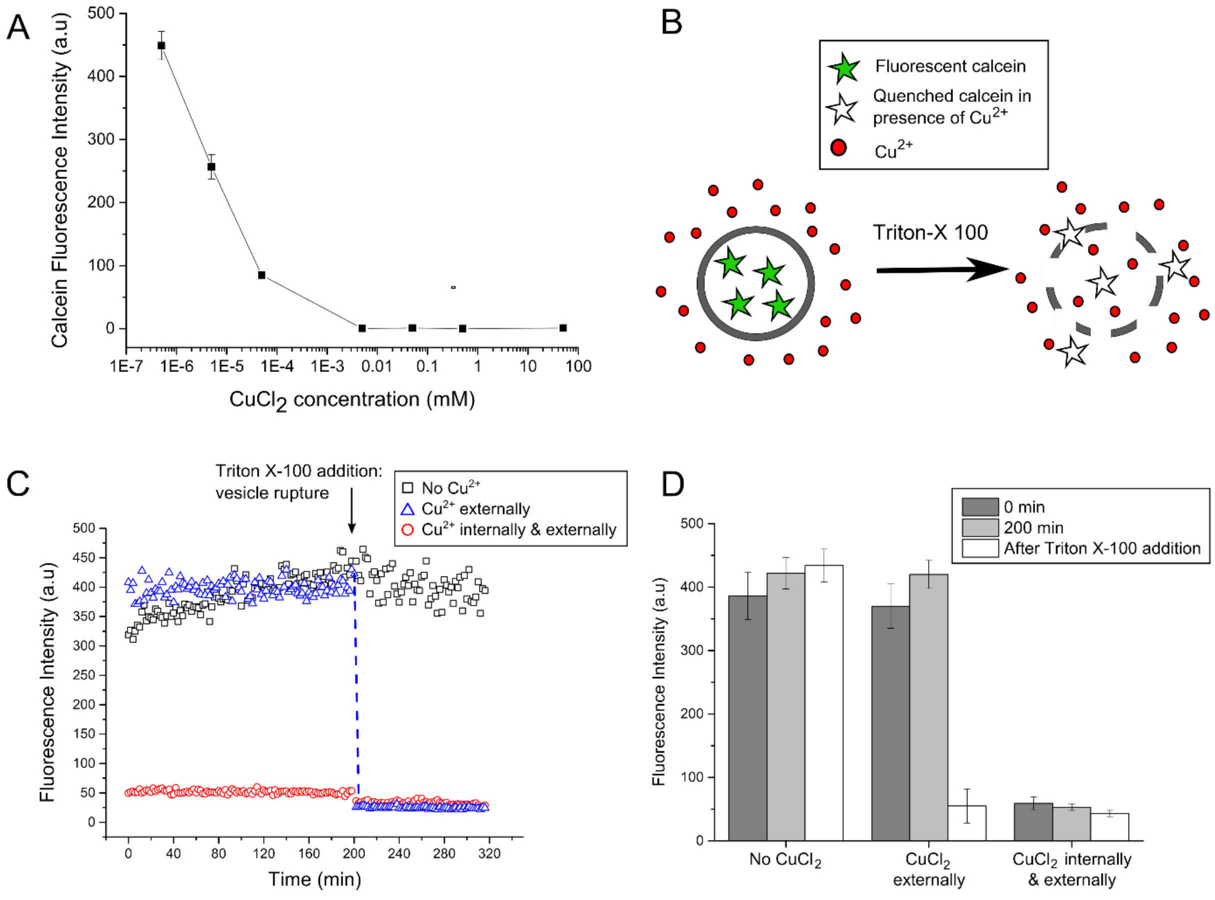

Figure SI 3. Cu^{2+} vesicle permeation assay. (A) Calibration curve showing quenching of calcein at 5 µM Cu^{2+} and above. Error bars = S.D from 5 independent trials. **(B)** Assay schematic. **(C)** Typical calcein fluorescence trace over time of calcein-containing GUVs in three conditions. Triton X-100 detergent was added at 200 min to rupture the vesicles. When Cu^{2+} was present externally, vesicle rupture led to immediate quenching (dotted blue line). The constant fluorescence before this point indicated lack of Cu^{2+} permeation to the GUV interior. **(D)** Average fluorescence at different time points in the three conditions tested. Error bars = S.D from five independent trials.

SI References

1 Yin, H. & Marshall, D. Microfluidics for single cell analysis. *Current opinion in biotechnology* **23**, 110-119 (2012).

2 Köster, S. *et al.* Drop-based microfluidic devices for encapsulation of single cells. *Lab on a Chip* **8**, 1110-1115 (2008).

3 Hu, P. C., Li, S. & Malmstadt, N. Microfluidic Fabrication of Asymmetric Giant Lipid Vesicles. *ACS Applied Materials & Interfaces* **3**, 1434-1440, doi:10.1021/am101191d (2011).

4 Hamada, T. *et al.* Construction of asymmetric cell-sized lipid vesicles from lipid-coated water-in-oil microdroplets. *The Journal of Physical Chemistry B* **112**, 14678-14681 (2008).

5 Ito, H. *et al.* Dynamical formation of lipid bilayer vesicles from lipid-coated droplets across a planar monolayer at an oil/water interface. *Soft Matter* **9**, 9539-9547 (2013).

6 Shen, A. Q., Wang, D. & Spicer, P. T. Kinetics of colloidal templating using emulsion drop consolidation. *Langmuir* **23**, 12821-12826 (2007).

7 He, M., Sun, C. & Chiu, D. T. Concentrating solutes and nanoparticles within individual aqueous microdroplets. *Analytical chemistry* **76**, 1222-1227 (2004).

8 Walde, P., Cosentino, K., Engel, H. & Stano, P. Giant Vesicles: Preparations and Applications. *ChemBioChem* **11**, 848-865, doi:10.1002/cbic.201000010 (2010).

9 Karamdad, K., Law, R., Seddon, J., Brooks, N. & Ces, O. Preparation and mechanical characterisation of giant unilamellar vesicles by a microfluidic method. *Lab on a Chip* **15**, 557-562 (2015).
10 Matosevic, S. & Paegel, B. M. Layer-by-layer cell membrane assembly. *Nature chemistry* (2013).
11 Matosevic, S. & Paegel, B. M. Stepwise synthesis of giant unilamellar vesicles on a microfluidic assembly line. *Journal of the American Chemical Society* **133**, 2798-2800 (2011).
12 Abate, A. R., Chen, C.-H., Agresti, J. J. & Weitz, D. A. Beating Poisson encapsulation statistics using close-packed ordering. *Lab on a Chip* **9**, 2628-2631 (2009).
13 Edd, J. F. *et al.* Controlled encapsulation of single-cells into monodisperse picolitre drops. *Lab on a Chip* **8**, 1262-1264 (2008).
14 Wu, L., Chen, P., Dong, Y., Feng, X. & Liu, B.-F. Encapsulation of single cells on a microfluidic device integrating droplet generation with fluorescence-activated droplet sorting. *Biomedical microdevices* **15**, 553-560 (2013).
15 Breuer, W., Epsztejn, S., Millgram, P. & Cabantchik, I. Z. Transport of iron and other transition metals into cells as revealed by a fluorescent probe. *American Journal of Physiology-Cell Physiology* **268**, C1354-C1361 (1995).
16 Fujii, S. *et al.* Liposome display for in vitro selection and evolution of membrane proteins. *Nat. Protoc* **9**, 1578-1591 (2014).
17 Thomas, J. M., Friddin, M. S., Ces, O. & Elani, Y. Programming membrane permeability using integrated membrane pores and blockers as molecular regulators. *Chemical Communications* (2017).
